# Effects of novel diabetic therapeutic footwear on preventing ulcer recurrence in patients with a history of diabetic foot ulceration: study protocol for an open-label, randomized, controlled trial

**DOI:** 10.1186/s13063-021-05098-8

**Published:** 2021-02-17

**Authors:** Yun Gao, Chun Wang, Dawei Chen, Hui Huang, Lihong Chen, Guanjian Liu, Shuang Lin, Min Liu, Xiaorong Wen, Jae-Hoon Cho, Yong Chen, Yingzhu Li, Xingwu Ran

**Affiliations:** 1 Diabetic Foot Care Center, Department of Endocrinology and Metabolism, West China Hospital, Sichuan University, 37 Guo Xue Xiang, Chengdu, Sichuan 610041 People’s Republic of China; 2 Department of Evidence-Based Medicine and Clinical Epidemiology, West China Hospital, Chengdu, 610041 People’s Republic of China; 3 Department of Ultrasound, West China Hospital, Sichuan University, Chengdu, 610041 People’s Republic of China; 4 Research Institute of DAJIM INC KOREA, Busan, 46721 Republic of Korea; 5 Health Research Center, Beijing OUNCE Health Technology CO., LTD, Beijing, 100021 People’s Republic of China; 6 Research & Development Center, Beijing OUNCE Health Technology CO., LTD, Beijing, 100021 People’s Republic of China

**Keywords:** Diabetic therapeutic footwear, Ulcer recurrence, Diabetic foot ulcers, Randomized controlled trial, Adherence to treatment

## Abstract

**Background:**

Recurrence after the healing of a foot ulcer is very common among patients with diabetes mellitus. Novel diabetic therapeutic footwear consisted of merino wool, vibration chip, and orthopedic insoles is designed to influence multifaceted mechanisms of foot ulcer occurrence. The aim of this study is to examine the effect of the optimally designed therapeutic footwear on preventing ulcer recurrence in patients with a history of diabetic foot ulcers (DFU).

**Methods/design:**

The trial is designed as a two arms, parallel-group, open-label randomized controlled intervention study. The Log-rank Test was used for calculating sample size based on the latest national multicenter survey data of DFU in China. Three hundred and twenty participants will be recruited from the Diabetic Foot Care Center, West China Hospital, Sichuan University. Adults with diabetic peripheral neuropathy, healed foot ulceration in the 3 months prior to randomization, and aged ≥18 years, will be recruited. Participants will be randomized to receive novel diabetic therapeutic footwear (*n* = 160) or their own footwear (*n* = 160). The primary outcome will be the incidence of ulcer recurrence. The secondary outcome will be measurements of barefoot dynamic plantar pressures, the influence of footwear adherence on ulcer recurrence, and the incidence of cardiovascular events. Assessment visits and data collection will be obtained at baseline, 1, 3, 6, 9, and 12 months. The intention-to-treat principle will be applied. A cox regression model will be used to calculate the hazard ratio for the incidence of ulcer recurrence. The change of barefoot dynamic plantar pressures will be assessed using repeated measures ANOVA. The study protocol has been approved by the Ethics Committee of The Biomedical Research Ethics Committee of West China Hospital, Sichuan University (Reference No. 2019(96)).

**Discussion:**

This clinical trial will give information on the ability of novel diabetic footwear on preventing ulcer recurrence in patients with a history of diabetic foot ulceration. If the optimally designed therapeutic footwear does work well, the findings will contribute to the development of innovative treatment devices for preventing foot ulcer recurrence in high-risk patients.

**Trial registration:**

Chinese Clinical Trial Registry ChiCTR1900025538. Registered on 31 August 2019.

**Supplementary Information:**

The online version contains supplementary material available at 10.1186/s13063-021-05098-8.

## Background

The development of foot ulcers is a significant marker of the extent of long-term complications in patients with diabetes mellitus, which is the most common reason for lower limb amputation [1]. Diabetes-related foot ulcers precede at least 60% of all nontraumatic lower limb amputations [2]. Mortality following amputation ranges from 50 to 68% at 5 years and is worse than that of many forms of cancer [2, 3]. Moreover, even after the resolution of a foot ulcer, recurrence is also common [4, 5]. A recent large cohort study in China showed that the incidence rate for foot ulcer recurrence within 1 year also reached 31.6% [5]. Therefore, prevention of ulcer recurrence or occurrence is of prime importance in the current approach to diabetic foot disease.

DFU is an outcome of a combination of multiple causes including pathophysiologic changes, foot deformities, and trauma [2]. Although pathophysiologic changes such as peripheral neuropathy or/and peripheral arterial disease (PAD) play a central role in the development of foot ulcers, foot deformities contributes directly to foot ulcer occurrence. These deformities as a result of diabetic autonomic and motor neuropathies lead to areas of increased peak plantar pressures, which are common sites of ulceration formation [2]. To reduce ulceration formation, diabetic therapeutic footwear for high-risk patients was widely recommended in clinical practice [6]. Reductions of plantar pressures, shear forces, and friction on the sole of the foot and the toes are primary mechanisms of therapeutic footwear, which are responsible for protecting against foot ulcers [6–16]. Data from observational studies suggested a significant protective benefit from improved custom-made footwear [7–12]. However, in well-designed randomized controlled trails (RCTs), no statistically significant benefit was observed in patients wearing improved footwear compared with those with usual care [13–15]. Based on several published reviews of clinical trials, the discrepancy between observational studies and RCTs might be attributed to characteristics of intervention shoes, adherence to treatment, risk factors for reulceration, and so on [15, 16]. Thus, footwear effectiveness may not only need a function of offloading but also adequate patients’ adherence [14–16].

**Fig. 1:**
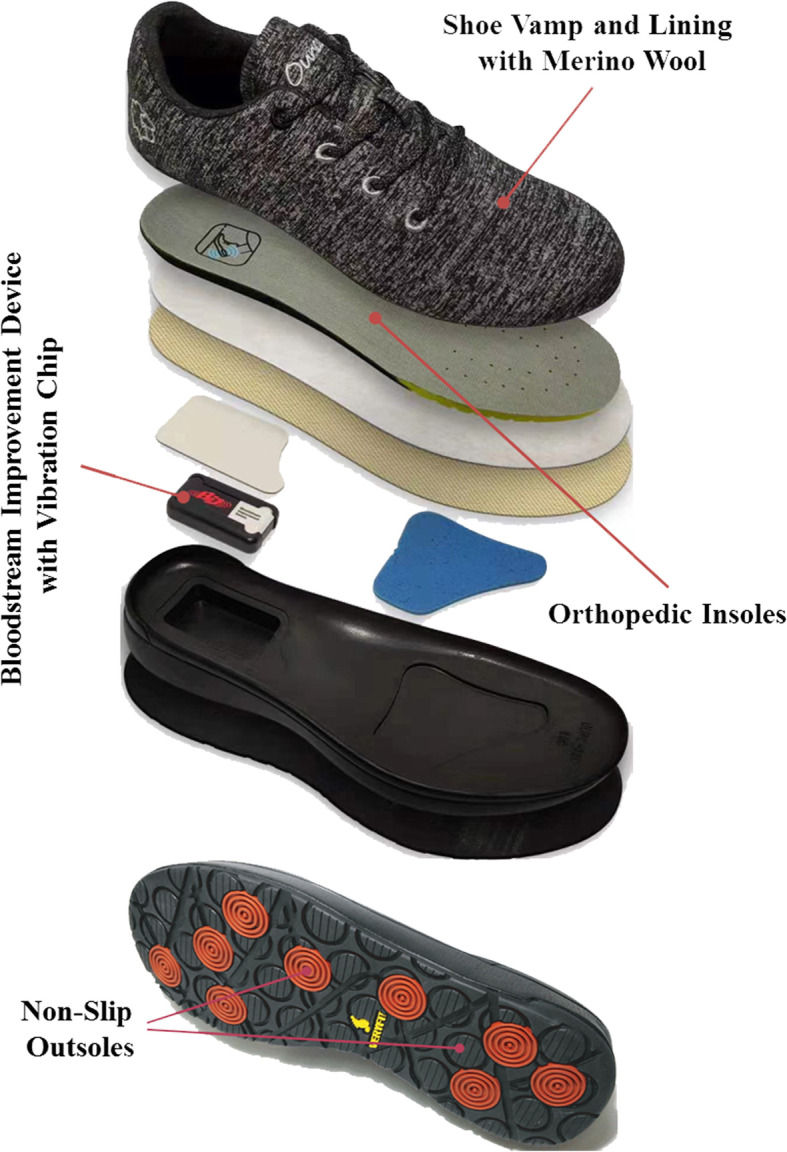
Key components of optimally designed therapeutic footwear

For better patients’ adherence and multifactorial intervention aimed at multiple risk factors for DFU, novel diabetic therapeutic footwear consisted of merino wool, vibration chip, orthopedic insoles, and non-slip outsoles is recently designed for use by patients with diabetes (Fig. 1). Merino wool is a high-quality type of wool which enables the production of lighter and softer wool fabrics with its long and fine fibers [17]. Shoe vamp and lining with merino wool had unique water absorption capacity, could adjust the temperature of the sole of the foot and produce a feeling of coolness or warmth, resisted odor and pathogenic bacteria, and was not easy to cause itchy skin, which could improve comfort and patients’ footwear adherence [18–20]. Among patients with diabetes, microvascular and macrovacular disease frequently contribute to reduced blood perfusion and skin oxygenation of feet which were independently related to foot ulcer risk. Therefore, the intervention to improve foot circulation may be beneficial to lower foot ulcer risk. Vibration chip was developed as a whole body vibration (WBV) exercise device. Prior studies have showed that WBV was beneficial to increase lower extremity skin blood flow and muscle force. Moreover, it also could improve the sensation of plantar surface pressure and standing balance [21–24]. In addition, whole body vibration intervention also could improve glycemic profile and lipid-related cardiovascular risk factors [25]. Additionally, previous literature has documented that the forefoot (e.g., metatarsal heads and toes) and heel with increased peak plantar pressures were most common sites of DFU [26]. To relieve the pressure on the forefoot and heel, orthopedic insoles will be designed to provide more support for medial longitudinal and lateral arch and partly transfer pressure away from the forefoot and heel. Moreover, they will employ soft and skin-friendly design to reduce the risk of friction-related skin breakdown. Therefore, in view of the above mentioned mechanisms, we speculate that the novel diabetic therapeutic footwear may exert beneficial effect on preventing foot ulcers in patients with diabetes. We will conduct a randomized controlled trial to examine the effect of the novel diabetic footwear with vibration chip and merino wool on preventing ulcer recurrence in patients with a history of DFU. Secondly, we further assess the influence of patients’ footwear adherence on the efficacy of the diabetic footwear.

## Objectives

The objectives of this trial are as follows:

1. To verify the efficacy of the therapeutic footwear with vibration chip and merino wool prevent ulcer in preventing ulcer recurrence among patients with a history of DFU.
2. To determine whether the therapeutic footwear can reduce the plantar pressure among patients with a history of DFU.
3. To examine whether patients wearing the therapeutic footwear have better feeling of comfort and higher footwear adherence and also assess the influence of patients’ footwear adherence on the efficacy of the therapeutic footwear.
4. To examine whether patients wearing the diabetic footwear have fewer cardiovascular events than those wearing non-improved footwear.

## Methods/design

**Fig. 2:**
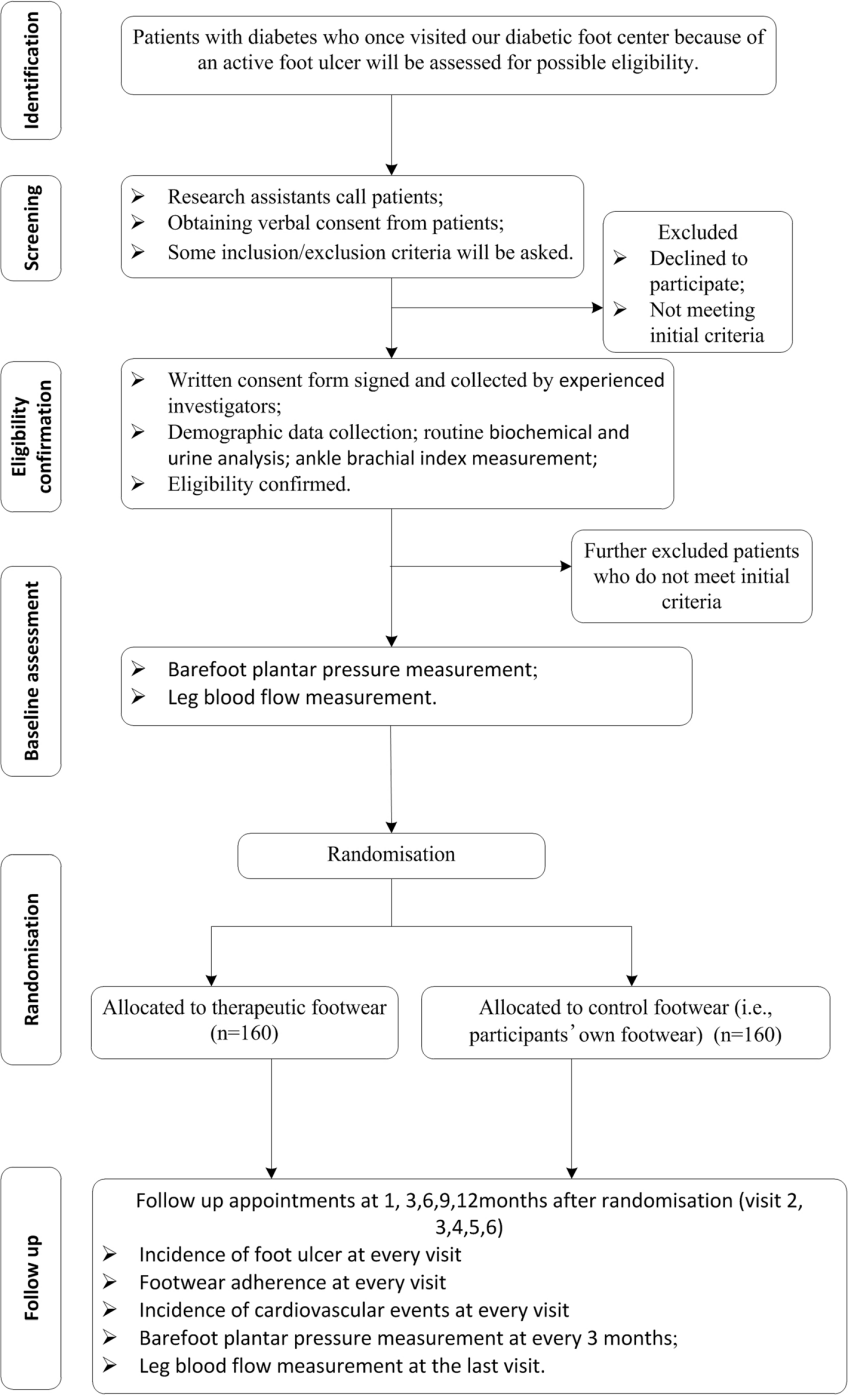
Trial flow chart

**Fig. 3:**
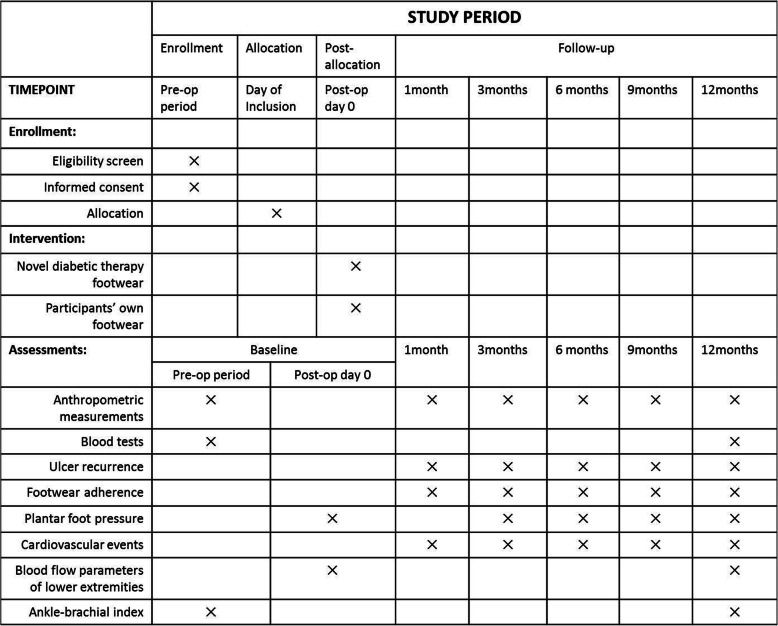
Schedule of enrolment, intervention, and assessment

The study was designed as a prospective, parallel group, single-blinded, randomized controlled clinical trial. The study protocol was written in accordance with the Standard Protocol Items: Recommendations for Interventional Trials (SPIRIT) guidelines (Additional file 1 shows the SPIRIT checklist). The trial has been registered in both English and Chinese at chictr.org.cn (ChiCTR1900025538) on August 31, 2019. The flowchart of the study design is shown in Fig. 2 and a detailed schedule of enrolment, intervention, and assessment is given in Fig. 3.

### Study site

All assessments will be conducted within the Diabetic Foot Care Center, Department of Endocrinology and Metabolism, West China Hospital, Sichuan University, Chengdu, Sichuan, People’s Republic of China. Our Diabetic Foot Care Center is one of largest multidisciplinary care centers in China with a database of more than 2500 patients with a history of DFU. Therefore, we would be able to recruit adequate eligible participants.

### Participants

Patients with a history of DFU whose healing time of foot ulcer was over 3 months will be invited to participate in our trial by phone. Individuals will be eligible for the trial if they meet the following inclusion criteria: (1) aged ≥ 18 years, (2) type 1 diabetes (T1DM) or type 2 diabetes (T2DM), (3) decrease or loss of protective foot sensation due to/as a result of diabetic peripheral neuropathy, and (4) had a healed foot ulceration in the 3 months prior to randomization. However, individuals will not be enrolled into this trial if they meet the following exclusion criteria: (1) amputation proximal to the tarsometatarsal joint, (2) the use of walking aids that offload the foot, (3) severe lower extremity artery disease (ankle brachial index ≤ 0.4, intermittent claudication or rest pain), (4) osteoarticular diseases unrelated to T1DM or T2DM, (5) decrease or loss of vision that is difficult to treat or correct, (6) unstable cardiovascular disease or unstable ischemic cerebrovascular disease, (7) severe renal dysfunction (estimated glomerular filtration rate (eGFR) < 30 mL/min per 1.73 m^2^) or severe liver dysfunction (Child-Pugh grade C), (8) malignant tumor, (9) severe illness that would make 24-month survival unlikely (as judged by the patients physician), (10) pregnancy or breast-feeding women, (11) neurologic or psychiatric disease that may prevent compliance with the protocol, and (12) patients who are considered unsuitable for the trial by investigators.

Potential participants will be given a brief verbal explanation of the trial by their treating podiatrist. Research assistants will give them telephone interviews and invite patients who are willing to participate to our diabetic foot center for further confirming eligibility and baseline measurements. Written informed consent will be obtained by a trained and experienced podiatric nurse or podiatrist at the baseline visit. On the consent form, participants will be asked whether they agree to use of their data and biological specimens when they choose to withdraw from the trial and asked for permission for the research team to share their relevant data with people from the universities taking part in the research or from the regulatory authorities.

### Sample size calculation

PASS 11.0 software was used to obtain sample size. As a parallel-group randomized controlled trial, the Log-rank Test (Lakatos) was used for the sample size calculation based on the following parameters or assumptions:

1. The survival probability of treatment group was estimated for 77.9%. According to the latest national multicenter survey data in China, 31.6% patients with a history of DFU developed new ulcer during 1 year follow-up [5]. Thus, the survival probability of control group was assumed to be 64.8%. We assume that the treatment group could have a 30% higher survival probability than control group.
2. The accrual period for patients’ recruitment was estimated for 12 months. The accrual pattern would be equal. The follow-up period would be 12 months. Therefore, the total time of this trial would last 24 months.
3. The power was set at > 0.8, while *α* was 0.05. The dropout rate of 0.2 was assumed. No switching between two groups. Two groups would have same sample size.

Therefore, the total sample size calculated using PASS was at least 314 cases. In this trial, we would recruit a total of 320 patients (160 for each group).

### Randomization and blinding

The randomization at the level of the individual will be performed by a statistician at the Chinese Evidence-based Medicine Center, West China Hospital, Sichuan University. SAS procedure PROC PLAN will be used to generate the randomization schedule. An independent offsite investigator who is not involved in the enrollment or assessment of participants will be responsible for keeping the randomization schedule in sealed envelopes. After the baseline assessment, the independent offsite investigator will open an opaque envelope sequentially and assign each eligible participant to the corresponding group.

Due to the special designation of the therapeutic footwear, it is not possible to blind participants to their allocated group. Thus, this trial will be an open-label clinical study and blinding will not be adopted. For reducing bias, participants will not be informed which footwear is intended to provide improved outcome. Moreover, every effort will be made to ensure that the primary outcome assessors and the statistician remain blinded to treatment allocation until the end of the study.

### Study procedures

All participants will be invited to attend the baseline visit and five further study appointments for 12 months on post-randomization. Data at every visit will be collected in case report forms (CRFs) by the trial team. All data will be entered into a secure database by research assistants (i.e., data collectors). In the current trial and further ancillary studies, there are no plans for collection, laboratory evaluation, and storage of biological specimens for genetic or molecular analysis.

#### Baseline visit

After signing an informed consent, participants will be interviewed using a standard questionnaire to obtain information on demographic characteristics (including sex, date of birth, race, education, occupation, annual income, residence, family history and diabetic duration), lifestyle risk factors (physical activity, smoking status and alcohol consumption), history of past and present illness, and current medications. Blood pressure, height, weight and waist circumference will be measured with the use of standard methods. Fasting blood samples and urine sample will be collected to test blood routine, liver and renal function, eGFR, glucose, lipid, serum creatase, glycated hemoglobin (HbA1c), urine routine, and urinary albumin to creatinine ratio. Ankle brachial index (ABI) will also be measured using a pulse waveform analyzer BP-203RPE II (form PWV/ABI, Omron Healthcare Co., Kyoto, Japan).

Based on the above-mentioned information, eligible participants will be defined by an on-site investigator and then randomized to the study group or control group by an independent offsite investigator. Subsequently, every randomized participant will continue to perform plantar pressure tests by the trained podiatrist. Footscan® Pressure Measurement System (RSscan international NV, Paal, Blegium) will be used to obtain the data of plantar pressure. Before plantar pressures are measured, each participant will undertake at least two practice walks along the plate walkway to familiarize them with the experimental process. Then, each participant will be asked to walk in bare feet upon the footscan platform at self-selected gait speed for at least three successful trials. The foot data including peak plantar pressure will be recorded by the Footscan® pressure measure system, which automatically divided the foot into 10 masked regions: hallux, toes 2–5, first to fifth metatarsals, midfoot, medial heel, and lateral heel. Locations of previous ulceration, foot deformity or preulcerative signs will be the primary sites of interest. In additional, blood flow parameters of lower extremities, which include maximum systolic velocity, maximum diastolic velocity, and time-averaged mean velocity, will also be assessed by a vascular ultrasound specialist in the vascular ultrasound room of our hospital that is a quiet temperature-controlled environment. Measures will be performed at the same time of day for individual subjects.

#### Follow-up visits

Participants will be followed at 1 month (visit 2), 3 months (visit 3), 6 months (visit 4), 9 months (visit 5), and 12 months (visit 6) after randomization. At each visit, the trial team will collect the information including incidence of foot ulcer, footwear adherence, cardiovascular events, glucose profile, and other cardiovascular risk factors control. For physical activity evaluation, participants will be asked to wear a pedometer (P084, SPORTWAY, ShenZhen, China) every day to record the date and steps taken per day. Meanwhile, a concealed activity monitor (Lifecorder Plus, Suzuken) will be attached to the diabetic therapeutic footwear. Activity data will be uploaded by a research assistant to a centralized server via internet at each follow-up visit. Adherence to treatment will be evaluated through the comparison of the step counts recorded by a concealed activity monitor with those recorded by a pedometer. Adherence to treatment will be monitored until the end of follow-up. Plantar pressure distribution patterns will be measured after randomization every 3 months. At the end of follow-up, ABI and blood flow parameters of lower extremities will also be collected. At each visit, if participants make a withdrawal request or are not eligible to continue due to the presence of severe concomitant diseases or conditions listed in the exclusion criteria, they will be discontinued from the study. For participants who drop out, the information including incidence of foot ulcer, footwear adherence, cardiovascular events, ABI (if possible), plantar pressures (if possible), and biochemical parameters (if possible) will be collected.

To improve participant retention and complete follow-up, the following measures will be adopted: participants’ contact information as well as contact information for at least two persons who would know the participants’ whereabouts and could get messages to them will be collected from the patients; a certain amount of subsidies (e.g., RMB 200) will be given to compensate participants for transportation and other costs at every follow-up visit; participants will receive phone appointment 1 day before every follow-up visit. If a participant could not be reached, a research assistant will make other appointments for the participant at least three times on different days, in an effort to schedule the site visit; a social networking platform (e.g., WeChat) will be used to connect with participants and then help investigators know participants’ condition in time, which will contribute to improve participants’ adherence and reduce lost to-follow-up.

### Intervention

Two different types of footwear will be evaluated for feasibility and acceptability in this trial: optimally designed therapeutic footwear (Fig. 1) and usual care (patients’ own footwear for control group).

#### Optimally designed therapeutic footwear

Therapeutic footwear with merino wool, vibration chip, and orthopedic insoles will be optimally designed by the Beijing Ounce Health Technology Limited Company. The size of footwear will be defined based on patients’ foot size. Merino wool from New Zealand will be used for the manufacture of comfortable shoe vamp and lining. A Vibration chip is included in a Bloodstream Improvement Device (BID) which is designed to change external impact energy into vibration energy and placed at the back of the heel of therapeutic footwear. BID is a magnetic field generated by repulsive force that does not require any electric devices and can operate semi-permanently. The vibration of BID is natural penetration vibration penetrating deeply into the human body, which is a nature-friendly vibration different from other battery vibration. Orthopedic insoles will be designed and optimized using the footscan® system (RSscan international NV, Paal, Blegium). The insole will be constructed from merino wool and high-density polyurethane foam materials. Characteristics of orthopedic insoles and shoe soles were showed in Table 1.

**Table 1:** Characteristics of orthopedic insoles and shoe soles of novel diabetic therapeutic footwear

Orthopedic insoles	
Rigidity	38 shore C hardness
Material	Polyurethane
Thickness of forefoot area	6 mm
Thickness of medial arch areas	12 mm
Thickness of lateral arch areas	8 mm
Thickness of heel area	6 mm
Shoe soles	
Thickness of forefoot area	12 mm
Heel height	23 mm
Midsole	
Rigidity	60 ± 3 shore C hardness
Material	Polyurethane
Specific gravity	0.9
Outsole	
Rigidity	60 ± 3 shore A hardness
Material	Rubber
Specific gravity	1.1
Thickness	4 mm
Depth of non-slip pattern	2.5 mm
Abrasion resistance	295 NBS
Tension resistance	142 kg/cm

#### Control footwear

The control group will receive patients’ own footwear. Control footwear will be constructed from materials commonly used in the manufacture of athletic shoes.

### Outcome measures

#### Primary outcome measure

The main study outcome measure is the incidence of ulcer recurrence. Ulcers are defined as cutaneous erosions through the dermis that may extend to involve the subcutaneous tissue or even to the level of muscle or bone [27, 28]. We expect at least a 30% reduction in the incidence of ulcer recurrence from baseline to the end of intervention.

#### Secondary outcome measures

The secondary outcome measures include (1) the change of barefoot dynamic plantar pressures, from the third to the sixth diaries compared with that from the first diary (screening period); (2) the influence of footwear adherence on ulcer recurrence; and (3) the incidence of cardiovascular events.

### Data management

All researchers including investigators, data collectors (i.e., research assistants), study coordinators, site monitors, data managers, outcome assessors, and a statistician will receive special training regarding the standard procedure and data management. During the recruitment period, data collectors will afford the collection of the baseline characteristics of participants and record them on study-specific CRFs. Data collectors will also collect and record the reports of laboratory findings and outcome measures on the CRFs during the recruitment or follow-up periods. Data will then be entered into Excel spreadsheets or EpiData manager by two collectors independently. Outcomes will be assessed by the principal investigator blind to intervention allocation during the study period. In the course of the trial, a data manager will be assigned to manage the relevant data. Researchers will assure that any personal information of participants collected remains strictly confidential. All data will be identified using participant numbers instead of directly displaying participant’s personal information. Access to the information will be restricted to the principal investigator or to researchers directly involved in the research at all times, before, during, and after the research activities. The data manager will check all data recorded on the CRFs and electronic documents after every visit and report back to the principal investigator and data collectors if there is any discrepancy. All data will be managed based on the Data Protection Act of 1998 [29]. All hard copy documents related to the research will be saved in a locked filing cabinet of Good Clinical Practice (GCP) Center of West China Hospital while electronic documents will be stored in a special computer with protected password. All research documents will be saved for at least 5 years after publication. If readers have any questions about our published data, they could contact our corresponding author (i.e., the principal investigator) to obtain the original data.

### Statistical analysis

At the end of this trial, all original data without participants’ name and the random sequences will be sent to a statistician for data analysis. Statistical analysis will be performed using SAS version 9.4 (SAS Institute Inc., Cary, NC, USA). Significance level will be set at *α* = 0.05. Quantitative variables expressed as means ± standard deviation or median with interquartile range, and categorical data will be recorded as numbers and percentages. Between-group differences of continuous variables will be tested for significance using Student *t* test when original data or data via transformation of variables is normally distributed or using Wilcoxon rank-sum test when data is not normally distributed. A chi-square test will be used for categorical data.

The primary end point, ulcer recurrence, will be analyzed according to the intention-to-treat principle, with event curves for the time to the first event on the basis of Kaplan–Meier analysis, and treatments will be compared by means of the log-rank test. A cox regression model will be used to calculate the hazard ratio for the primary end point. Among secondary end points, plantar pressures at baseline and at 3, 6, 9, and 12 months will be compared with repeated measures ANOVA. The changes blood flow parameters of lower extremity at baseline and at 12 months will be compared using Student *t* test when these means between groups are comparable at baseline or using analysis of covariance (ANCOVA) when these means are not equivalent at baseline. For cardiovascular events, the hazard ratio for the comparison of treatment group with control group and 95% confidence intervals will be estimated with the use of a proportional-hazards model.

The footwear adherence will be calculated for each group and then ulcer recurrence rates will be compared between the subgroups of patients with high adherence and low adherence by *χ*^2^ tests. We also plan to explore whether potentially important covariates such as age, sex, body mass index, physical activity, and diabetic duration could potentially confound the results.

## Discussion

Over the past two decades, whether therapeutic footwear prescribed for patients with diabetic foot was effective to prevent ulcer recurrence remains controversial.

Epidemiological studies showed that therapeutic footwear was associated with decreasing ulcer recurrence [7–12]. However, well-designed RCTs have failed to produce the expected outcome [13–15]. The therapeutic footwear in previous RCTs mainly contributed to the relief of mechanical pressure. In the present study, however, the therapeutic footwear encompass a number of new technologies (showed in Fig. 1) to reduce high vertical or shear stress of feet, improve distal lower-extremity blood delivery and intensify patients’ comfort [7, 18, 21, 22]. As a multifaceted intervention for causes of foot ulcers, we speculate that the therapeutic footwear in this study could exert beneficial influence in preventing ulcer recurrence. At the end of the trial, we will compare the results of this study with those of previous RCTs. For the inconsistent results between the present and previous studies, we will further analyze possible reasons that lead to these discrepant outcomes of different studies. Moreover, the correlation between outcomes and new technologies will also be assessed in the present study.

High plantar pressures associated with the neuropathic foot increase the risk of foot ulcer development [30]. The use of foot shape with barefoot plantar pressure measurements in designing custom insoles has been documented to significantly decrease forces on high-pressure areas under the forefoot in patients with diabetes [31] and thus could prevent foot ulcer development [4]. Despite the absence of custom insoles, the therapeutic footwear used in this trial contains orthopedic insoles which can provide more support for medial longitudinal and lateral arch and partly transfer pressure away from the forefoot and heel. In a preliminary test with 99 patients with diabetes at high risk for foot ulceration, we assessed the effect of the therapeutic footwear on barefoot plantar pressures. The result showed that the barefoot dynamic plantar pressures were significant lower after using the therapeutic footwear for about 6 weeks compared with before use (172.67 ± 15.53kpa vs. 177.07 ± 15.71kpa, *p* = 0.049) (unpublished data). This may be attributed to orthopedic insoles of the therapeutic footwear. Thus, we consider that the change of barefoot dynamic plantar pressures could be used to assess the efficacy of the therapeutic footwear on plantar pressures. Whole body vibration (WBV) training is a relatively new form of exercise. Studies have showed that therapeutic WBV could increase skin blood flow and whole blood NO concentrations and improve standing balance in patients with diabetic neuropathy [21, 24]. Moreover, WBV was beneficial to improve glycemic profile, lipid-related cardiovascular risk factor, and leg blood flow among patients with T2DM [21, 25]. In this trial, the orthopedic insoles and BID with vibration chip, which are two key components of the therapeutic footwear, will play the above roles. Thus, we will validate if the special designed therapeutic footwear could achieve the desired effect through the measurements of barefoot dynamic plantar pressures and blood flow velocity of lower extremity.

In addition, adherence to treatment has been considered as an important factor to influence those clinical outcomes of RCTs [14, 15]. Patients who were adherent had significantly better outcomes than those who were nonadherent [14, 15]. Thus, we will ensure patients fully understand the importance of adherence to treatment through continuous education and meanwhile repeatedly urge patients to persist footwear adherence during the process of trial. Moreover, we also focus on identifying patients who are nonadherent or are anticipated to be nonadherent. After this trial is completed, the effect of footwear adherence on the outcomes on ulcer recurrence will be evaluated and then whether new technologies influence footwear adherence will also be assessed.

The main limitation of the study is the open-labeled design and single-institutional study. The patients’ preference and expectations from trial participation with a specific footwear therapy may lead to bias. However, due to the obviously different structures between therapeutic footwear and control footwear, it is difficult to blind participants to their allocated group. Participants will not be informed which footwear is under investigation for its capacity to protect against ulcer recurrence. I will also conceal participants’ allocation to outcome assessors and a statistician in order to try to minimize bias. Additionally, we are not able to purchase a reliable in-shoe pressure measurement system within China. Although the change of in-shoe plantar pressures is important, the measurement of in-shoe pressures is unavailable for us to examine the efficacy of the therapeutic footwear. Even so, the lack of in-shoe pressure measurement should have limited impact on assessing the effect of therapeutic footwear because ulcer recurrence is the primary outcome in the present trial.

## Ethics and dissemination

The study will be completed in accordance with the ethical principles in the Declaration of Helsinki and the International Conference on Harmonization (ICH) GCP Guideline. Chengdu Science and Technology Bureau and West China Hospital, Sichuan University are the trial sponsors and will monitor and audit this project to ensure compliance with the necessary legislation.

This is a low risk trial because therapeutic footwear is a non-invasive treatment which will be delivered by a trained, registered podiatric nurse or podiatrist. Any adverse events will be recorded by investigators and reported to the trial team. Participants will also be offered the opportunity to report any adverse events at follow-up assessments.

At the end of the trial, we will present our findings at the national and international conferences and peer-reviewed journals of medicine.

## Trial status

This protocol is the first version, which approved on April 11, 2019. Recruitment of participants will commence on March 2021. Recruitment will be stopped automatically as soon as 320 patients have been included, which will be completed approximately on March 2022.

## Supplementary Information

**Additional file 1.** SPIRIT 2013 Checklist: Recommended items to address in a clinical trial protocol and related documents.

## Data Availability

Not applicable

## Abbreviations

DFU: Diabetic foot ulcers
PAD: Peripheral arterial disease
RCTs: Randomized controlled trails
WBV: Whole body vibration
eGFR: Estimated glomerular filtration rate
T1DM: Type 1 diabetes
T2DM: Type 2 diabetes
CRFs: Case report forms
HbA1c: Glycosylated hemoglobin
ABI: Ankle brachial index
BID: Bloodstream Improvement Device
GCP: Good clinical practice
